# A Reliable Protocol for *In situ* microRNAs Detection in Feeding Sites Induced by Root-Knot Nematodes

**DOI:** 10.3389/fpls.2016.00966

**Published:** 2016-07-07

**Authors:** Fernando E. Díaz-Manzano, Marta Barcala, Gilbert Engler, Carmen Fenoll, Janice de Almeida-Engler, Carolina Escobar

**Affiliations:** ^1^Área de Fisiología Vegetal, Facultad de Ciencias Ambientales y Bioquímica, Universidad de Castilla-La ManchaToledo, Spain; ^2^Institut Sophia Agrobiotech, Université de Nice-Sophia AntipolisSophia Antipolis, France

**Keywords:** nematodes, *Meloidogyne* spp., galls, giant cells, *in situ* microRNAs, tomato, miR390

## Abstract

Galls induced by *Meloidogyne* spp. in plant roots are a complex organ formed by heterogeneous tissues; within them there are 5–8 giant cells (GCs) that root-knot nematodes use for their own nurturing. Subtle regulatory mechanisms likely mediate the massive gene repression described at early infection stages in galls, particularly in giant cells. Some of these mechanisms are mediated by microRNAs (miRNAs); hence we describe a reliable protocol to detect miRNAs abundance within the gall tissues induced by *Meloidogyne* spp. Some methods are available to determine the abundance of specific miRNAs in different plant parts; however, galls are complex organs formed by different tissues. Therefore, detection of miRNAs at the cellular level is particularly important to understand specific regulatory mechanisms operating within the GCs. *In situ* hybridization (ISH) is a classical, robust and accurate method that allows the localization of specific RNAs directly on plant tissues. We present for the first time an adapted and standardized ISH protocol to detect miRNAs in GCs induced by nematodes based on tissue embedded in paraffin and on-slide ISH of miRNAs. It can be adapted to any laboratory with no more requirements than a microtome and an optical microscope and it takes 10 days to perform once plant material has been collected. It showed to be very valuable for a quick detection of miRNAs expression pattern in tomato. We tested the protocol for miR390, as massive sequencing analysis showed that miR390 was induced at 3 dpi (days post-infection) in *Arabidopsis* galls and miR390 is 100% conserved between *Arabidopsis* and tomato. Successful localization of miR390 in tomato GCs constitutes a validation of this method that could be easily extended to other crops and/or syncytia induced by cyst nematodes. Finally, the protocol also includes guidance on troubleshooting.

## Introduction

MiRNAs are short [20–24 nucleotides (nt)], non-coding RNAs that are important components of gene regulatory plant networks (Liu and Chen, 2009; Axtell, 2013; Bologna and Voinnet, 2014; Borges and Martienssen, 2015), with roles in gene silencing at transcriptional and post-transcriptional levels. So far, 34 miRNA families have been described in plants that are strictly associated with plant development and involved in processes such as cell proliferation, nodule, and lateral root development (Jin et al., 2013). The complexity of the interrelationships in the regulatory process involving miRNAs is hindering rapid and effective progress in this field. Current roles of most miRNAs in plants remain unclear, particularly in non-model species. Many of the proposed functions are just projections based on homology with known miRNAs from other species (Rhee and Mutwil, 2014).

An important step in understanding the role of a miRNA is to identify the tissue and cell type within which it is expressed. However, miRNAs abundance is predominantly analyzed using whole plants or organs, while many of them are expressed only in few cell types. A clear example in the context of general gene expression profiles came from the comparison of the transcriptomes of both entire galls (that contain GCs) and isolated GCs (giant cells) that were strikingly different, and a strong dilution of the GC-specific transcripts was observed when whole galls were analyzed (Barcala et al., 2010; Portillo et al., 2013; Cabrera et al., 2014). Therefore, the study of regulatory sRNAs that might be exclusive for GCs formation and/or maintenance from whole-gall RNA samples can be very arduous, as frequently the analysis of the whole organ fails to attribute the proper cell-specific functions to a gene. Such questions have led to the development of methods that allow scientists to examine the expression of specific genes in particular cells (Cabrera et al., 2014, 2015); by cell isolation techniques or *in situ* localization, e.g., in nematode feeding sites (NFS) (Portillo et al., 2009; Szakasits et al., 2009; Barcala et al., 2012; Anjam et al., 2016). Up to date no protocol for miRNAs localization in GCs has been described, thus, here we present an adapted protocol to localize miRNAs from galls and their corresponding control (uninfected roots).

Plant transformation with promoter::reporter constructs of miRNAs could be an option to study their activation patterns at cell/tissue level, but generating the appropriate transgenic plants in crops such as tomato is time consuming and it is not totally equivalent to detect miRNA abundance. Galls are a mixture of heterogeneous tissues, within them, the GCs experience mitosis and polyploidization (reviewed in de Almeida-Engler and Gheysen, 2013; Escobar et al., 2015). Therefore, it is important to distinguish the specific tissues and/or cells where a particular sRNA/miRNA is expressed within the gall. The isolation of single cells, such as GCs involves specialized and expensive equipment such as a micro-aspirator or a laser microdissector (Portillo et al., 2009; Szakasits et al., 2009; Barcala et al., 2012; Anjam et al., 2016). However, common assays for detecting cell-specific expression patterns, like ISH, can be applied to different plant species and transgenic plants in different organs or single cells.

Protocols for the *in situ* detection of mature miRNAs in plants are available (Várallyay and Havelda, 2011) and some of them were improved after using LNA (Locked Nucleic Acids). These are modified DNA probes that increase considerably the hybrid stability (Javelle and Timmermans, 2012; Yao et al., 2012). We adapted those protocols and minimized the time for fixation, inclusion, and hybridization of miRNAs in roots infected by endoparasitic nematodes that lead to NFS formation (galls) using double-labeled LNA probes. The presented protocol is an efficient and reasonably fast method to study cell-specific expression patterns of miRNAs in tomato with putative roles during gall formation and/or its maintenance. Besides, we consider that the protocol could be modified with minor changes to other vegetable crops species e.g., those resilient to transformation.

## Protocol overview

**Figure 1:**
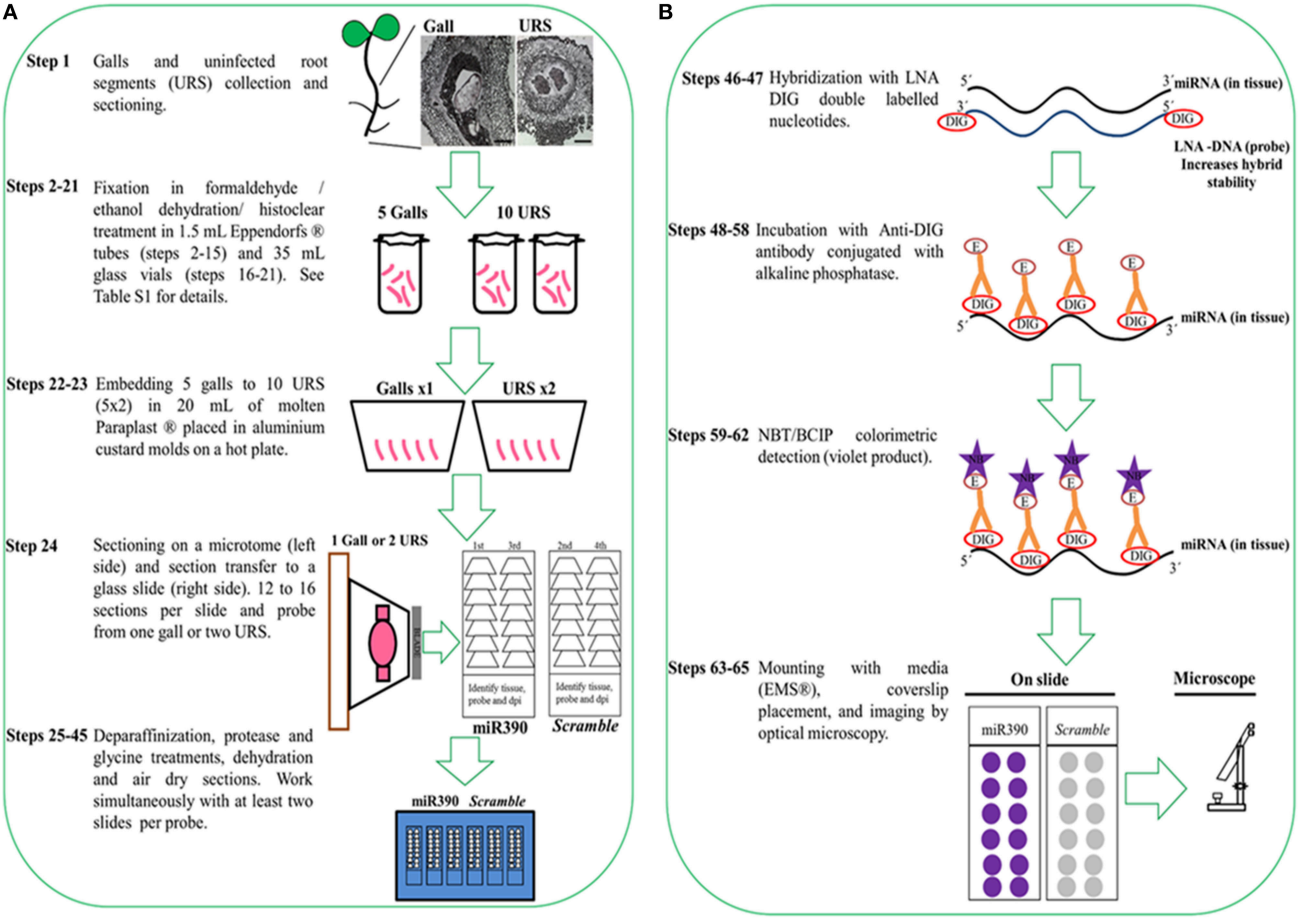
**Protocol flowchart**. Schematic diagram of ISH procedure representing all necessary steps from sample collection to colorimetric detection of miRNAs. The whole procedure takes 10 days. **(A)** Plant tissue is fixed in formaldehyde/ethanol solutions/histoclear, followed by embedding in Paraplast® X-tra and sectioning. **(B)** An anti-DIG antibody conjugated with alkaline phosphatase and its substrate is used for the detection of the DIG labeled products. DIG, digoxigenin; E, enzyme; NB: NBT/BCIP violet product.

**Figure 2:**
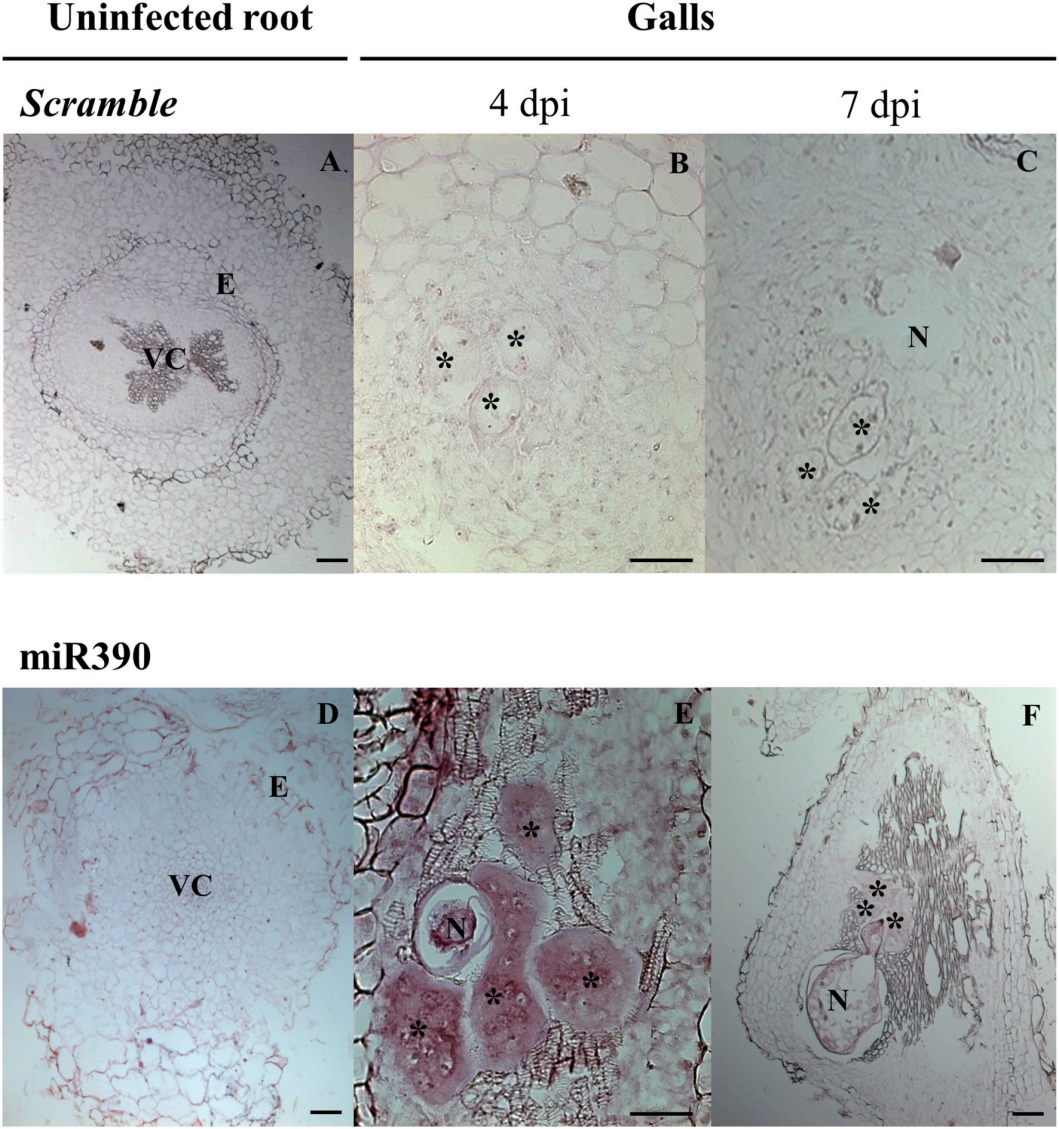
*****In situ*** detection of mature miR390 expression in tomato galls. (A-C)** *Scramble* was used as a negative control, alongside a miR390 specific probe **(D-F)**. **(A,D)**, uninfected root segments (URS); **(B,E)**, *Meloidogyne incognita* galls at 4 dpi, **(C,F)**, galls at 7 dpi. Asterisks indicate giant cells; N, nematodes; VC, vascular cylinder; E, endodermis. Scale bar: 50 μm **(B,E)**; 100 μm **(A,C,D,F)**.

To preserve the morphology of the plant tissue (galls) and the stability of miRNAs, fresh tissue samples (nematode infected and uninfected roots) are collected and instantly fixed in formaldehyde by sequential vacuum infiltration pulses of the fixative (Figure 1; Supplementary Table 1). The plant tissue is dehydrated in an ethanol series and then embedded in Paraplast® X-tra (see reagents, number 26) for subsequent classical sectioning on a microtome. Sections (ribbons) are collected on coated slides (Thermo Scientific SuperFrost™; see Supplementary Image 1). Protease treatment is performed to eliminate cellular RNases and protein excess (step 34, Supplementary Table 1) which would otherwise interfere with the signal. MiRNA hybridization and antibody incubation are followed by subsequent washing steps (steps 46–56, Supplementary Table 1). Optimization of anti-DIG temperature is essential for the successful *in situ* localization of small RNAs (miRNA detection treatments). In our case, temperatures around 37°C have improved detection efficiency of miRNAs during the hybridization with 5′ and 3′ double DIG-labeled miRCURY LNA™ miRNA detection probes (Exiqon® A/S^1^; Vedbaek, Denmark) in contrast to previous described miRNA protocols (Várallyay and Havelda, 2011; Javelle and Timmermans, 2012; Yao et al., 2012), reducing the time consumed and increasing the detection of the miRNA. The sections are incubated with an anti-DIG antibody conjugated to alkaline phosphatase which binds to the DIG-labeled miRNAs (Figure 1B). Adding specific substrates for alkaline phosphatase allow the colorimetric detection of the DIG-labeled miRNAs products. At this point of the protocol, the slides containing the sections are observed under a microscope (Figure 1B). Those with well-preserved tissues are selected, mounted, labeled, and visualized with bright field optics (Figure 2).

### Plant growth and tissue sampling

While this protocol focuses on the detection of miRNAs from root tissues and galls in tomato (*Solanum lycopersicum*), we think that it can be easily adapted to other crop species with minor changes during fixation and embedding. Plant roots grown in soil or agar should be immersed repeatedly in sterile water, using a soft paintbrush to remove particulate matter while minimizing harm to the roots. It is important to consider the stage of plant growth and tissue type since gene expression may differ. We sampled galls and uninfected root segments (URS) from plants at seven days post-germination. Time should be minimized between collection and fixation to avoid degradation of target miRNA while enough URS and gall tissue should be used for hybridization with each probe (Figure 1A). Three independent experiments are recommended.

### Tissue preparation

The proposed method for tissue preparation involves formaldehyde fixation, ethanol dehydration, Histo-Clear® (see reagents, number 17) clearing, and paraffin embedding. It is a reasonably fast and simple procedure that conserves sufficiently tissue morphology, while preserving miRNAs. We reduced sample fixation time in formaldehyde to around 14 h at 4°C (Fox et al., 1985); contrary to classic mRNA ISH which may fix longer than 1 week (de Almeida-Engler et al., 2001). Following formaldehyde fixation (steps 2-4, stage A, Supplementary Table 1), the tissue should be embedded in Paraplast® (see Supplementary Table 1 and Figure 1A for details). Images of Paraplast® sections (10 μm; Figure 1A) should be better defined than thicker sections, like those obtained from agarose-mounted specimens (100-300 μm).

We recommend limiting the number of galls to each mold so that liquid paraffin can polymerize between the samples (0.5 and/or 1 cm^2^ of tissue in 8 cm^3^ of paraffin; Figure 1A). Sectioning one paraffin block should yield enough tissue sections of multiple galls or URS to perform ISH of at least two to three miRNA probes. We suggest carrying out at least 12–16 sections per probe and per independent experiment corresponding to one biological replicate (gall or URS; Figure 1A, Supplementary Image 1) to check that the expression pattern is consistent within the tissues. Simultaneously, a negative control probe is recommended with equivalent or correlative sections from the same paraffin block. Consequently, sufficient sections for each probe plus its negative control (24–32 sections; Supplementary Image 1) of galls and URS should be used for hybridization (Figure 1B) as they might be damaged during the procedure. It is also important to check the integrity of the tissues and the homogeneous adherence to the slides under the microscope after sectioning, since the tissue may separate from the paraffin or detach from the slides during the procedure (stage C; Supplementary Table 1).

### Probe design

Double-labeled LNA modified oligonucleotide probes were used during the detection stage, solving the problem of a low annealing temperature that would be required by the small probe length (see Table 1) as they enable high hybrid stability. Therefore, hybridization can be performed at 50°C (see step 46, Supplementary Table 1). Double-labeling of probes at both ends also facilitates a more intense signal, making detection easier and increasing significantly the signal resolution and sensitivity, thus radioactive ^35^S-labeled probes can be circumvented (de Almeida-Engler et al., 2001). We used LNA™ probes from Exiqon®, but another option is to make your own customized probes. Oligonucleotides should be 18–24 bases long and they should have an annealing temperature of ~70°C. Using a negative control probe is highly recommended, preferably one that does not match any miRNAs in the available web databases. Herein, we used *Scramble* from Exiqon® (see Table 1), a probe with no hits or >70% homology to any sequence in any organism in the NCBI database and no homology to sequences in the miRBase database. Many pre-designed specific probes for known miRNAs are available together with negative control probes. The specific mature probe detected here is *sly-miR390b-5p* from tomato (MIMAT0035479), and it is 100% homologous to that from *Arabidopsis* (Table 1). The method confirmed the expression of the miR390 in tomato.

**Table 1:** **Probe sequences: list of mature miRNAs for tomato (***sly, Solanum lycopersicum***) with the highest homologies to the Exiqon® probe (40143-15) used**.

**MiRNA probe used**	**Reference from Exiqon^®^**	**Probe sequence**	**Accession number**	**Mature miRNA**	**Mature sequence in tomato according to miRBase**	***E*-value**
MiR390	40143-15	GGCGCTATCCCTCCTGAGCTT	MIMAT0035479	*sly-miR390b-5p*	AAGCUCAGGAGGGAUAGCGCC	0.002 ^*^
MiR390	40143-15	GGCGCTATCCCTCCTGAGCTT	MIMAT0035467	*sly-miR390a-5p*	AAGCUCAGGAGGGAUAGCACC	0.008
MiR390	40143-15	GGCGCTATCCCTCCTGAGCTT	MIMAT0035468	*sly-miR390a-3p*	CGCUAUCCAUCCUGAGUUUUA	0.320
MiR390	40143-15	GGCGCTATCCCTCCTGAGCTT	MIMAT0035480	*sly-miR390b-3p*	CGCUAUCCAUCCUGAGUUUCA	0.320
*Scramble*	99004-15	GTGTAACACGTCTATACGCCCA	No matches were found	Not found	Not found	Not found

*The sly-miR390b-5p bears a 100% homology to the probe (asterisk). Source: [http://www.exiqon.com/microrna-In-situ-Hybridization-detection-probes] and [http://www.mirbase.org]*.

### Optimization of ISH conditions

Before hybridization of any plant tissue (e.g., *Arabidopsis*, corn, potato, rice, tomato, etc.), probe concentration, hybridization temperature and buffer concentrations need to be optimized to maximize the sensitivity of the *in situ* experiment. It is then essential to test different concentrations of each probe in order to optimize signal specificity. Therefore, it is recommended to perform an initial experiment to determine the most appropriate concentration of the probe to be used. Herein, we tested different concentrations of the probes (5, 10, 15, and 20 nM) and hybridization temperatures (40, 48, 50, and 55°C; data not shown). In our hands, one of the best concentrations was 20 nM for tomato paraffin sections to maximize discrimination of miRNA abundance between tissue types (infected vs. URS) for miR390. It is recommended to use the lowest possible hybridization temperature that still does not produce any background signal to minimize damage into tissue samples. We found that 50°C was the most appropriate temperature according to the probe used, hybridization conditions and sections preservation (see step 46, Supplementary Table 1).

### Validation and controls

To validate the expression pattern for the miRNA of interest, we routinely performed multiple technical replicates of at least two slides containing multiple sections. Herein, it is recommended to follow the protocol with a total of 24–32 paraffin sections per independent biological sample; 1 gall or 1-2 URS (Supplementary Image 1). A negative control (here, *Scramble*) is also used in half of the 24–32 sections for absence of signal since it should not show specific miRNA hybridization. It is highly recommended to include it alongside each tissue type analyzed (galls and URS; Supplementary Image 1, Figures 2A-C). Another recommended control is to omit the probe during the hybridization (step 46, Supplementary Table 1). The miRNA probe used in this study is also suitable as a positive control (miR390) for galls if other miRNAs are examined as it gives a clear positive signal in gall tissue at 4 to 7 dpi (Figures 2D–F). To tackle potential problems that may occur during the protocol, see Table 2; Supplementary Table 1.

**Table 2:** **Troubleshooting: list of practical recommendations during the whole procedure**.

**Problem**	**Steps**	**Possible reason**	**Way out**
Erratic staining in the positive control	1–24	RNA is degraded or contaminated	Decrease time of fixation, dehydration and/or inclusion and be careful in the RNA handling
Tissue fragility-incomplete Paraplast® embedding	Microtome sections	The long axis of the sample is not perpendicular to the blade on the microtome	Observe the first 4–8 sections obtained: if they are streaked or ribbons are not formed, adjust the orientation of the block to 10°. Remove the Paraplast® mold and ensure that the side stuck on the wood block is perfectly flat
Very weak signal	34	High protease-RNAse treatment	Extended incubation times and higher protease concentrations can increase signal strength, but over digestion will lead to tissue damage and reduced signal intensity
	54	Antibody concentration	Increase the incubation temperature or concentration of antibody up to four times
Non-hybridization signal is detected	46	High hybridization temperature	Lower the temperature of the hybridization
		Low probe concentration	Increase the probe concentration
High background of hybridization signal that it is not present in the negative control	46	Low hybridization temperature	Raise the temperature to improve the specificity of the hybridization
Signal loss and/or morphology	48–49	High concentration of SCC buffer.	Decrease the concentration of SSC buffer and increase formamide concentration
The tissue is not properly stacked onto the slide	55–58	Washing buffer in excess	Reduce washing time
Saturated staining	59	NBT/BCIP overdose	Diminish overall staining time

*The specific points of the protocol are indicated*.

## Materials and equipment

### Reagents

1. Anti-DIG-AP, Fab fragments from sheep (Roche®, cat. no. 1 093 274; store at 4°C).
2. Blocking reagent (Roche®, cat. no. 1 096 176; store at 4°C).
3. Bovine serum albumin (BSA; ≥98%) (Sigma®, cat. no. A7906; store at 4°C).
4. Deionized formamide (Sigma®, cat. no. F9037; store at 4°C). Toxic and hazardous; wear gloves for protection; and discard them properly.
5. Denhardt's® solution 50x (Sigma®, cat. no. D2532; store at –20°C).
6. DEPC (Diethylpyrocarbonate; Sigma®, cat. no. 77017; store at 4°C). Harmful and hazardous; wear gloves for protection; and discard them properly.
7. Dextran sulfate (Sigma®, cat. no. D8906-5G; store at 4°C).
8. DMF (*N,N*-Dimethylformamide; Sigma®, cat. no. D4551). Toxic and hazardous; wear gloves for protection and dispose of properly.
9. Double-labeled probes can be ordered directly from Exiqon®. It is recommended to order 5′ and 3′ double DIG-labeled LNA-modified probes.
10. DTT (Dithiothreitol; Sigma®, cat. no. D9779).
11. EDTA (Ethylenediaminetetraacetic acid; Sigma®, cat. no. E9884).
12. Eosin (Sigma®, cat. no. 230251).
13. Ethanol (100%) and dilution series in water (VWR®, cat. no. 20821.330).
14. Formaldehyde (40%; Sigma®, cat. no. F8775; store at 4°C). Toxic and hazardous; wear gloves for protection and discard them properly.
15. Glycine (Sigma®, cat. no. G7403).
16. HCl (Chlorhydric acid; Sigma®, cat. no. 258148). Toxic and hazardous; wear gloves for protection and dispose of properly.
17. Histo-Clear® (Fisher Scientific®, cat. no. 50-899-90147).
18. *In situ* mounting medium (EMS®, cat. no. 17988-30; store at 4°C).
19. KCl (Potassium chloride; Sigma®, cat. no. P9541).
20. KH_2_PO_4_ (Potassium dihydrogen phosphate; Sigma®, cat. no. P0662).
21. NaCl (Sodium chloride; Sigma®, cat. no. S9888).
22. Na_2_HPO_4_ (Disodium hydrogen phosphate; Sigma®, cat. no. S7907).
23. NaH_2_PO_4_ (Sodium dihydrogen phosphate; Sigma®, cat. no. S8282).
24. NaOH (Sodium hydroxide; Sigma®, cat. no. 221465).
25. NBT/BCIP stock solution (Roche®, cat. no. 1 681 451; store at 4°C).
26. Paraplast® X-tra (Sigma®, cat. no. P3808).
27. Protease from *Streptomyces griseus* (Pronase E; Sigma®, cat. no. P5147; store at −20°C).
28. RNase-free H_2_O (Life® Technologies, cat. no. 10977023).
29. Sodium acetate (Sigma®, cat. no. S2889).
30. Sodium citrate (Sigma®, cat. no. 71497).
31. Sodium phosphate (Sigma®, cat. no. 342483).
32. Tris base (Tris(hydroxymethyl)aminomethane; Sigma®, cat. no. 252859).
33. Triton X-100 (Sigma®, cat. no. T8787).
34. Tween-20 (100%; Duchefa®, cat. no. P-1362).
35. tRNA (Roche®, cat. no. 109541; store at −20°C).
36. Ultrapurified H_2_O (e.g., Milli-Q; Millipore).

### Reagent setup

All reagents should be prepared in advance unless explicitly specified. Solutions must be stored at RT in the dark except otherwise is indicated.

1. Anti-DIG solution (freshly prepared before use): dilute anti-DIG-AP to 0.6 U mL^−1^ (0.8 μL anti-DIG per 1 mL) in washing buffer.
2. Blocking buffer (freshly prepared before use): mix 1% (wt/vol) of blocking reagent in 1x TBS buffer pH 7.5. Heat 200 mL of TBS up to 70°C, add 2 g blocking reagent and dissolve by stirring constantly while the solution cools down gradually to RT.
3. DEPC treated Milli-Q H_2_O: add 1 mL of DEPC in 1L of Milli-Q H_2_O. Either Milli-Q water or DEPC treated Milli-Q water can be used to prevent RNases activity.
4. Dextran sulfate (100% wt/vol): dissolve 5 g of dextran sulfate in 4 mL of Milli-Q water. Stir overnight at 4°C; the next day, adjust volume to 5 mL with Milli-Q H_2_O. Filter through a 0.45 μm size pore filter and aliquot into 1 mL Eppendorf® tubes. Store at −20°C.
5. DMF (70%): mix 0.7 mL of 99% DMF and 0.3 mL of Milli-Q H_2_O. Store in 1 mL aliquots at −20°C.
6. DTT (1 M): mix 5 g of DTT in 32.5 mL of 10 mM sodium acetate (pH 5.2) and pass through a 0.22 μm filter. Store in 1 mL Eppendorfs® at −20°C.
7. Double DIG- labeled LNA™ 5′ and 3′ probes: use 100 μL per slide (20 μL probe plus 80 μL hybridization buffer). Dilute the probe with 50% deionized formamide at the required concentration (20 nM) and store at −20°C for no longer than 6 months.
8. Eosin (0.1%): dissolve 0.1 g eosin Y in 0.1 L of 90% ethanol by gently stirring. Store at 4°C.
9. Formaldehyde (4%): under a fume hood, mix 169 mL of 1x PBS buffer, 100 mL of Milli-Q H_2_O, 30 mL of 40% formaldehyde, 300 μL of 1 M DTT, 300 μL of Tween-20 (100%), and 300 μL of 100% Triton X-100. Store at 4°C.
10. Glycine (10% wt/vol): for stock concentration, dissolve 10 g of glycine in 100 mL of Milli-Q water and pass it through a 0.22 μm filter; store at 4°C. For working concentration, dilute to 0.2% by mixing 2 mL in 98 mL of 1x PBS. Store at 4°C.
11. HCl (1 M): under a fume hood, mix 8.4 mL of 37% HCl with 91.6 mL of Milli-Q H_2_O.
12. Hybridization buffer: combine 5 mL of 100% (vol/vol) deionized formamide, 2.5 mL of 100% (wt/vol) dextran sulfate, 1.25 mL of 20x ISH salts, 250 μL of 50x Denhardt's® solution, 125 μL of tRNA 100 mg mL^−1^, and 875 μL of Milli-Q H_2_O. Mix thoroughly and store at −20°C until use.
13. ISH salts (20x): prepare by mixing 4 M NaCl, 200 mM Tris-HCl (pH 7.5), 100 mM sodium phosphate (pH 6.8), and 100 mM EDTA. Store 1 mL aliquots at −20°C.
14. NaCl (0.1 M; 1x): prepare 4 M concentration by add 233.76 g of NaCl in 1 L of Milli-Q H_2_O. Stir and dilute with Mili-Q water to 0.1 M.
15. NaOH (10 M): dissolve 40 g of NaOH in 100 mL of Milli-Q H_2_O.
16. PBS (10x): dissolve 80 g of NaCl, 2 g of KCl, 14.4 g of Na_2_HPO_4_, and 2.4 g of KH_2_PO_4_ in 800 mL of Milli-Q H_2_O, adjust pH to 7.0 with 1 M NaOH. Adjust volume to 1 L with additional Milli-Q H_2_O. Sterilize by autoclaving at 1 atm at 120°C for 20 min (the same for the remaining autoclaved reagents).
17. Probe mix (freshly prepared before use): to prepare each slide, mix 10 μL of deionized formamide, the appropriate amount of LNA probe (determined experimentally) and RNase-free distilled H_2_O up to 20 μL.
18. Protease: dilute the content of the vial (0.1 g per 2 mL of Milli-Q water) in RNase-free H_2_O (final concentration of 50 mg mL^−1^) and pre-digest the protease by incubating at 37°C for 4 h. Store aliquots of 650 μL at −20°C.
19. SSC (20x): dissolve 175.3 g of NaCl and 88.2 g of sodium citrate in 800 mL of Milli-Q H_2_O. Adjust the pH to 7.0 with a few drops of 1 M HCl. Adjust the volume up to 1 L and autoclave.
20. Staining solution (NBT/BCIP; freshly prepared under a fume hood before use): NBT (yellow compound): mix 15 mg of NBT in 0.35 mL of 70% DMF plus 0.15 mL of Milli-Q H_2_O. BCIP (white compound): mix 7.5 mg of BCIP in 0.50 mL of 70% DMF. Both stock solutions can be stored at −20°C for short periods. Add the two compounds (0.5 + 0.5 mL) to 49 mL of 1x TN buffer.
21. TBS buffer (1x): dissolve 6.06 g of Tris base and 8.76 g of NaCl in 800 mL of Milli-Q H_2_O. Adjust pH to 7.5 with 1 M HCl and make the final volume up to 1 L with dH_2_O and autoclave.
22. TE solution (1x; Stop buffer): mix 10 mL of 1 M Tris-HCl (pH 8.0) and 2 mL of 0.5 M EDTA (pH 8.0) with 988 mL of Milli-Q H_2_O and autoclave.
23. TN buffer (1x; Substrate buffer): mix 165 mL of 3 M NaCl (pH 7) and 50 mL of 10x Tris-glycine (pH 8.0). Adjust pH to 9.5 by adding ~ 5 mL of 10 M NaOH and 285 mL of DEPC Milli-Q H_2_O. Autoclave as described previously.
24. Tris-glycine (10x): mix 30.2 g of Tris base and 188 g of glycine (electrophoresis grade) in 700 mL of Milli-Q H_2_O. Adjust the pH to 8.0 with 1 M HCl and make up volume to 1 L with Milli-Q H_2_O. Store at 4°C.
25. T-RNA (100 mg mL^−1^): dilute the powder from one vial of tRNA in 1 mL of DEPC Milli-Q water; store aliquots at −20°C.
26. Washing buffer (1x; BSA wash, 1% wt/vol; freshly prepare on day of use): dissolve 5 g powered BSA in 440 mL of Milli-Q H_2_O by stirring. Add 50 mL of 10x PBS, 0.5 mL of 100% Triton X-100 and Milli-Q H_2_O (about 10 mL) up to 500 mL. Filter through 0.45 μm size pore filter. Aliquot in 50 mL Falcon tubes and store at 4°C.

### Equipment list

1. Aluminum custard cups (Cominter Paper, S.A., cat. no. 81347; 7.5 cm diameter × 4.5 cm depth).
2. Binocular stereomicroscope (Nikon® SMZ 1000 or similar).
3. Cover slips, 24 × 60 mm #1 (VWR®, cat. no. 48404-452).
4. Dry heating block at 85°C (Eppendorf® ThermoStat plus 2 mL, cat. no. 5353 040.130).
5. Electronic motorized rotary microtome (Microm® HM® 360 or similar).
6. Filters: use 0.45 μm (Sartorius, cat. no. 16555-K) size pore filters for viscous compounds and 0.22 μm (VWR, cat. no. 28145-477) for water compounds.
7. Fume hood (Astec® Microflow, cat. no. M50547).
8. Glass bottles, 500 mL (SIMAX®, cat. no. BM0500).
9. Glass bottles, 1L (SIMAX®, cat. no. BOT5310).
10. Glass slide cuvettes (EMS®, cat. no. 70312-20).
11. Glass slide rack, fitting glass slides (EMS®, cat. no. 70312-24).
12. Glass vials, 35 mL (Dismadel, cat. no. 120-5802).
13. Hot plate set at 40°C (Selecta® Plactronic, cat. no. 6156100).
14. Humidity chamber: place moistened Whatman® or similar paper at the bottom of a flat stainless steel box. Seal tightly with Parafilm M® (EMS®, cat. no. 62010-37).
15. Incubator set at 37, 50, and 58°C (Selecta®, cat. no. 2000210).
16. Mercury thermometer (Sigma®, cat. no. Z676381-1EA).
17. Microscope and bright field imaging (Nikon® Eclipse 90i Microscope with camera Nikon® DXM 1200C or similar).
18. Parafilm M® (Bemis®, cat. no. PM 996).
19. Plastic trays (V. M. Packaging & Home Appliance (P) Limited, cat. no. 27047:179044).
20. Slides, 26 × 76 × 1 mm (Thermo® Scientific SuperFrost®, cat. no. AA00008032E).
21. Water bath plate at 58°C (Selecta® Multiplaces, cat. no. 7471200).

### Equipment setup

1. Binocular: a Nikon® binocular SMZ 1000 model was used to facilitate carving of the paraffin molds and to obtain an isosceles trapezoid-triangular shaped resin mold (Figure 1A). It is also used to help during sample collection (see step 1, Supplementary Table 1).
2. Microtome: use a tungsten blade to section 10 μm thick paraffin slices with an incidence angle of 5–7°. Assemble the microtome according to the manufacturer's instructions. Fill the integrated ice bath with dry ice to maintain the temperature at ~ 4°C.
3. Microscope: a Nikon® Eclipse 90i with a Nikon® DXM 1200C camera was used to obtain bright field images following the company's guidelines.

## Stepwise procedures

The *in situ* miRNA hybridization method shows the localization of mature miRNA in their cellular environment. It allows a visual and qualitative comparison of miRNA abundance among tissues in the same section. Preserving RNA is critical, owing to ubiquitous natural RNases. These will quickly destroy both the target RNA in the cell and the RNA probe. They may be found on glassware, reagents and on the hands, clothes and saliva of the manipulator, etc. Therefore, handlers should ensure an RNase-free environment to prevent contamination that will lead to degradation of the probe and/or tissue RNA. To avoid the presence of RNases, we recommend wearing gloves throughout the procedure and using sterile tubes, a cleaned bench top and DEPC-treated Milli-Q water for solutions. A fume hood must be used for organic solvents (formaldehyde, Histo-Clear®, and deionized formamide). To sterilize materials (scissors, tweezers and pipettes), you can use specific commercial sprays for inhibiting RNase action or autoclaved material at 120°C. Similarly, we use filter-containing pipette tips. Therefore, be careful at each protocol step to avoid RNase contamination.

The procedure must allow probe penetration while simultaneously preserving the tissue during the intense manipulation. Tissue preservation during fixation, dehydration, and inclusion (steps 1-24, stage A, Supplementary Table 1) is critical. Thus, we recommend that from the ethanol series until incubation in Histo-Clear®-paraffin and inclusion in pure paraffin the procedure should not take longer than 8 days.

## Stages of the protocol

The presented protocol was divided in five main stages: (A) Fixation and embedding of plant tissue in paraffin; (B) Sectioning and microscope pre-selection; (C) Deparaffinization and ISH of miRNA with DIG double-labeled LNA probes; (D) Detection and development with colorimetric alkaline phosphatase substrates; and (E) Assembly and image capture (see Supplementary Table 1 and Timing overview).

## Stage A. fixation and embedding in paraffin (see Supplementary Table 1)

### Time: 8 days

Buffers used for this stage should preferably be stored in the dark at 4°C. However, they can be maintained at RT and darkness for short-term storage (less than 1 month). Periods longer than 1 month stored at 4°C.

Particular attention when handling the tissue must be taken to avoid damage to tissue morphology. Prolonged tissue fixation and dehydration (more than 3 days) (see Supplementary Table 1, steps 1–15) can decrease the intensity of the miRNA signal. We recommend minimize interruptions during these steps of the protocol.

Important: prepare these reagents in advance (see reagents setup):

**Table:** 

**Day**	**Reagents to prepare**
1	1x PBS and 4% formaldehyde (w/v)
2	1x PBS and 10–50% ethanol/1x NaCl series (vol/vol)
3	70–85% ethanol/1x NaCl series, 90% ethanol/0.1% eosin, 95% ethanol/Milli-Q water and 100% ethanol (vol/vol)
4	100% ethanol, 100% ethanol/Histo-Clear® series, 100% Histo-Clear® (vol/vol) and add enough Paraplast® resin to melt
5	Histo-Clear®, 50% Histo-Clear®/Paraplast® (vol/vol) and molten Paraplast®
6–8	Molten Paraplast®

## Key notes to keep in mind during stage A (fixation and embedding, see Supplementary Table 1):

1. Sample collection (step 1): using a binocular, scissors and tweezers, carefully collect the tissue (galls and URS; see Figure 1A) and place it in a labeled 1.5 mL Eppendorf® tube, pre-filled with 1x PBS on ice.
2. Sample/buffers ratio (steps 1-3): we advise to collect each gall (~0.5 cm^3^ of tissue) and URS (1 cm^3^ of control tissue: lateral root primordia and/or root apex) in 1 mL buffer solution (1x PBS).
3. Fixative vacuum infiltration steps (steps 2-3): when making the formaldehyde fixation solution (use fume hood), place the tube containing the samples with the lids open and applied vacuum for 30 s. Release gently the vacuum for 5 min before repeating the vacuum infiltration step once more (steps 2–3, Supplementary Table 1). This will provide optimal formaldehyde infiltration and will allow air removal from samples (they will sink in the tube). Incubate samples at 4°C overnight in 4% formaldehyde (step 4).
4. Ethanol series (steps 6-15): ethanol solutions should be freshly made with NaCl 1x and Milli-Q water, respectively. Store at 4°C.
5. Eosin staining (step 11): for easy identification of the samples within the Paraplast® molds, you can stain the tissue with a mild non-interfering dye. The tissue should adopt a color sufficiently strong as to be located in the solutions from step 12. Sample color is usually pink-fuchsia, sometimes yellowish.
6. Paraffin embedding (steps 16-24):
  - Inclusion steps (steps 16-21) should be carried out in glass vials from the Histo-Clear® steps on, because reagents may damage plastic tubes (Várallyay and Havelda, 2011). See Supplementary Table 1 for volume details.
  - Melt in advance enough Paraplast® to use in steps 20-23 (see Supplementary Table 1). In an incubator at 58°C, a 500 mL bottle full of paraffin chips will take at least 12 h to melt homogeneously.
  - To prepare the 50% Histo-Clear®/Paraplast® (vol/vol), mix equal volumes of melted paraffin and Histo-Clear®. To prevent Paraplast® (wax) solidification (steps 20-21); preheat Histo-Clear® at 58°C in an incubator.
  - To manipulate samples during hardening (galls and URS; step 22), use flamed tweezers to avoid wax (Paraplast®) solidification.
  - Use clean and clearly labeled vials for each solution. Use sequentially 100% ethanol/Histo-Clear® (steps 16-18); 100% Histo-Clear® (step 19); 50% Histo-Clear®/50% Paraplast® (step 20); and 100% Paraplast® (steps 21-23).
7. Aluminum molds (steps 22 and 23):
  - To include the samples in Paraplast® resin, we use aluminum custard-like cups as molds. Each aluminum mold holds 5 samples (see Figure 1A).
  - First, place samples in glass vials (at 58°C) in molten paraffin (step 21) on a hot plate at 58°C (step 22).
  - Subsequently, samples will be placed in the molds filled with Paraplast® by using flamed tweezers. The precise placement of the samples within the Paraplast® mold is important for determining their orientation for longitudinal or transverse sections. Here, we chose longitudinal for both galls and URS.
  - Prepare a bath with iced water to transfer the molds into, this will allow the paraffin to polymerize smoothly (step 23).

At this point of the protocol, you can stop and store the samples for a long period, as they are embedded in paraffin. Blocks can be stored in the dark. RNases are not active while the samples are within the paraffin resin.

## Stage B. microscopy: sectioning and microscope pre-selection (see Supplementary Image 1)

### Time: 5 h per sample (gall and/or URS)

High quality sections are very important for good results.

## Key notes to keep in mind during Stage B (sectioning and microscope pre-selection; see Supplementary Image 1)

1. Sections of 10 μm are recommended for combining a proper signal and satisfactory tissue visualization. For sectioning, the use of an electronic retractable motorized microtome with a loupe attached will facilitate visualization of sectioned samples.
2. It is important to carve a trapezoid-triangular shape in the resin mold using a blade and place it in the microtome in a parallel orientation to the knife-edge plane (see Figure 1A). The trapezoid-triangular shaped mold will reduce the aggression by blade cutting. Use new tungsten blades, brushes and tweezers for handling sections to minimize RNase action or clean used ones with anti RNase product (For example, RNaseZap® RNase Decontamination solution; https://www.thermofisher.com/order/catalog/product/AM9780).
3. Fix the paraffin block onto a wood block in the microtome by slightly melting the paraffin placed on a metal knife with a lighter.
4. Paraffin sections are placed on SuperFrost® Menzel Gläser®^2^ slides (Supplementary Image 1). Sections are placed alternatively in two slides. In this way, correlative sections will be hybridized with either the specific probe or the control oligonucleotide.
5. Sections should be handled with tweezers or brushes when placed on glass slides. For easier manipulation, sections are placed on Milli-Q that was previously poured over the slides. Each slide should contain as many sections as possible, arranged in two columns. Similarly, we will separate replicates of each tissue to be tested by each probe on different slides. It is recommended to identify type of tissue, sample age and probe; use a pencil or a diamond pen (see Supplementary Image 1).
6. To avoid loss of material during next stage, it is crucial to keep the slides containing sections on a hot plate at 40°C overnight, so that the paraffin sections adhere well onto the slides. Check the morphological quality of sections under a microscope. Some slides can be also stained (e.g., toluidine blue) to assess tissue quality before use.
7. Choose slides with the best tissue morphology for hybridization. This will be crucial for precise signal localization. At least two to three slides per probe should be used per independent experiment (see Figure 1A).

## Stage C. hybridization with LNA double labeling (see Supplementary Table 1)

### Time: 1 day

Probes based on LNA modified oligonucleotides may be purchased from Exiqon®, labeling intensity will depend on the abundance of the target miRNA. For good signal in tomato plants, it is advisable to use 20 nM of 5′ and 3′ double-DIG labeled LNA probes. It is important to use at least one negative control, either an oligonucleotide that does not hybridize with any known miRNA and/or a similar sample processing treatment in the absence of probe to monitor non-specific background. In this study, we used the first option with an oligonucleotide called *Scramble*; see Figure 2 and Table 1.

Important: prepare the following reagents before starting (see reagents setup):

**Table:** 

**Steps 25–47**	**Reagents to prepare**
Dewaxing	100% Histo-Clear®
Hydration	100% ethanol, 95% ethanol/Milli-Q water, 75-10% ethanol/1x NaCl series (vol/vol) and 1x PBS
RNase treatment and washing	Prewarmed TE buffer at 37°C with 50 mg/mL protease, 0.2% Glycine in 1x PBS
Dehydration	10-75% ethanol/1x NaCl series (vol/vol), 95% ethanol/Milli-Q water and 100% ethanol
MiRNA hybridization	Two probes (miR390 and *Scramble*) at the required concentration (in this protocol, 20 nM). Set a dry heating block at 85°C
Detection and development	Warm up the 0.2x SSC buffer and the 0.2x SSC + 20% deionized formamide to the hybridization temperature (50°C)

## Key notes to keep in mind during Stage C (deparaffinization and hybridization; see Supplementary Table 1)

1. Dewaxing sections (step 25): to deparaffinize the tissue sections, treat with 100% Histo-Clear® for 4 min with gentle stirring. When the paraffin is removed from the slides, start rehydrating them with the ethanol/Milli-Q and ethanol/1x NaCl series (steps 26-32, Supplementary Table 1). We use sterile glass slide cuvettes.
2. Ethanol/1x NaCl series (steps 29-32 and 38-41, Supplementary Table 1) and PBS buffer series (steps 33 and 36-37; Supplementary Table 1): at this point one must be careful because the tissue is exposed again to the action of RNases.
3. Protease treatment (step 34): add 125 μL protease (50 mg/mL) in 50 mL 1x TE buffer to digest cellular RNases and protein excess.
4. 0.2% Glycine (step 35): add 2 mL of 10% glycine in 98 mL 1x PBS, this step will block protease activity.
5. Prehybridization drying (step 45): ensure the slides are dry before adding the hybridization solution.
6. Hybridization (step 46, Supplementary Table 1): heat the probe mix (final concentration 20 nM, 20 μL per slide) for 3 min at 85°C. Quickly place the probe mix on ice to prevent reassembly of nucleic acids and add 80 μL of hybridization buffer per probe, per slide. Total volume of hybridization solution per slide should be 100 μL (20 μL probe mix plus 80 μL of the hybridization buffer). Mix gently, to avoid the formation of bubbles. Place the probe mix on the edge of the slide and extend it with the coverslip (as if you were brushing without touching the sections) so that the sections are embedded with the hybridization mix. Then finally place gently the coverslip on the sections to assure that all tissue is in contact with the hybridization solution.
7. Slides are incubated in a stainless steel box containing a damp paper at the bottom surface. Seal the box around the edges with Parafilm M® before incubating at 50°C (step 46, Supplementary Table 1) to prevent that slides may dry.

## Stage D. detection and development (see Supplementary Table 1)

### Time: 1 day

Immunodetection of the miRNA (here miR390): once ISH is performed, the next step is incubation with the blocking solution, washing, and detecting the miRNA of interest in galls and URS.

Important: prepare the following reagents in advance (see reagents setup and Supplementary Table 1 for volume details):

**Table:** 

**Steps 48–62**	**Reagents to prepare**
Coverslip removal	Prewarm 0.2x SSC buffer and 0.2x SSC buffer + 20% deionized formamide at 50°C (step 48)
Washes	0.2x SSC buffer + 20% deionized formamide, 1x PBS and 1x TBS (steps 49-51 and 57)
Stop hybridization and detection	Blocking buffer (step 52), anti-DIG buffer (step 54) and washing buffer (steps 53, 55-56)
Raising tissue pH	1x TN (step 58)
Development	NBT/BCIP staining solution (step 59)
Treatment stop buffer	1x TE (step 60)

## Key notes to keep in mind during stage D (detection and development, see Supplementary Table 1)

1. All detection and development steps: should be done without stirring and in darkness. The same steel box used during the hybridization should be used through the next steps, since it keeps slides moist and in darkness.
2. Post-hybridization washes (step 48, Supplementary Table 1): rinse the slides gently to remove coverslip in 0.2x SSC (1 min) at the hybridization temperature (50°C). In the second 0.2x SSC wash (5 min), add 20% deionized formamide (step 49, Supplementary Table 1) at 50°C to prevent loss of tissue morphology.
3. Blocking, washing, and anti-DIG buffer (steps 52-54, Supplementary Table 1): slides are placed in the steel box and treated with the different buffers by using a 1 mL pipette.
4. TN buffer (step 58): incubate slides in 1x TN for 5 min to raise the pH to 9.5, this step allows optimal alkaline phosphatase activity.
5. Development (step 59, Supplementary Table 1): add the NBT/BCIP mix (see reagents setup, number 20) and monitoring under an optical microscope depending on how fast the signal comes up. Stop the reaction when the negative controls begin to show a light purple color. At this point the miRNA of interest should show a darker stain than the controls. The reaction is then stopped with 1x TE buffer. Here, galls and tomato roots were incubated for 22 h at RT, regularly monitoring it in order to detect when the signal for miR390 was evident.

## Stage E. mounting slides and imaging (see Supplementary Table 1)

### Time: 30 min per slide

Slides can be mounted in water for a quick check before adding the stop buffer. If signal is not yet strong enough, it can be developed further. Otherwise, proceed to mounting steps; dry well the slides before adding the mounting medium (see reagents, number 18) that is not water miscible. A coverslip should be placed on top of the sections, observe and register images under a bright field microscope.

## Key notes to keep in mind during stage E (mounting and photographing; see Supplementary Table 1)

1. Mounting medium: add to the slide by placing a line-mounting with a pipette in the middle of the slide. When the coverslip is placed, it should completely cover all the samples on the slide without the presence of bubbles (see step 63, Supplementary Table 1).
2. Dry mounted slides before observation to prevent that coverslip moves damaging the samples. It is recommended to leave the samples to dry overnight at RT (see step 64, Supplementary Table 1) or dry for 1 or 2 h at 37°C.
3. Pictures taken with bright field optics (see Figure 2) should include the complete gall (10-20x; see step 65, Supplementary Table 1).

## Results and discussion

Here, we present an efficient and improved protocol for miRNA ISH and their localization in root feeding sites induced by endoparasitic nematodes (de Almeida-Engler et al., 2012). We tested tomato galls induced by *Meloidogyne incognita*. Our previous data obtained from massive sequencing of small RNAs present in galls from *Arabidopsis* as compared to URS showed that miR390 was consistently induced in galls at early infection stages compared to uninfected roots (Cabrera et al., 2016). However, galls are pseudo-organs containing a mixture of heterogeneous tissues (reviewed in de Almeida-Engler and Gheysen, 2013; Escobar et al., 2015) with many differentially expressed genes, e.g., those involved in heat-shock (Escobar et al., 2003; Barcala et al., 2008); cell cycle (de Almeida-Engler et al., 1999, 2009); lateral root development (Cabrera et al., 2014, 2015); etc. Transcript abundance and transcriptional profiles are different in GCs compared to the rest of the gall tissues (vascular neighboring cells and cortical cells; reviewed in Escobar et al., 2011; Portillo et al., 2013). Hence, it was crucial to develop a method for the localization of miRNAs in cells and/or tissues within the gall where a particular miRNA is expressed and compare its relative abundance to uninfected controls. The expression pattern of miR390 has been studied during lateral root formation and in galls, using transgenic plants with reporter genes fused to the promoters of miR390 genes, such as *pMIR390a/b:GUS* (Marin et al., 2010; Cabrera et al., 2016). However, ISH of this particular miRNA has not been performed yet. MiR390 is 100% conserved among several plant crops species, such as *Cucumis melo, Oryza sativa, Solanum lycopersicum, Solanum tuberosum, Vitis vinifera, Zea mays*, etc. according to miRBase (see The MiRBase database^3^) and Supplementary Table 2 (marked with asterisks). We aimed to localize it in tomato galls and compared to uninfected roots.

A clear ISH signal for miR390 was localized for first time in tomato GCs at 4 and 7 dpi (Figures 2E,F, respectively) using a specific double-labeled LNA probe. Negative control with *Scramble* did not show any signal or background on either URS (Figure 2A) or gall tissues (Figures 2B,C). The specific signal was more intense at 4 dpi than at 7 dpi in galls and a low signal was found in URS (Figure 2D), what is essentially in agreement to the results of sRNA sequencing, where abundance of miR390 was high at 3 dpi and to the promoter activation of miR390a at 4 dpi. However, it seems that miR390 abundance decrease at 7 dpi, but the promoter of miRNA390a is still active at this infection stage (Cabrera et al., 2016). These results indicate that although the miR390 is present in uninfected tissues, it is more abundant in GCs within the gall than in the rest of the tissues. It is important to point that there are other techniques available to analyze gene expression specifically in nematode feeding cells, such as microarray analyses after microaspiration or laser micro-dissection (e.g., Szakasits et al., 2009; Barcala et al., 2010; Portillo et al., 2013). However, the amount of RNA extracted is normally very low and small RNA sequencing (sRNA-seq) protocols should be adapted, thus no record yet is available with the combination of both techniques (cell isolation plus sRNA-seq) in the plant-nematode interaction. In addition, those techniques do not discriminate among the different gall tissues, but detect sRNAs in an heterogeneous mixture of all gall tissues as a whole. Therefore, *in situ* hybridization is recommended to analyze the presence of miRNAs in the different gall tissues, as well as in the uninfected root tissues.

Although, the ISH experiments presented herein yielded reliable results, several problems were encountered during the course of protocol optimization. Among these, lack/weak hybridization signal or overstaining (data not shown; see Table 2 for troubleshooting guidelines) owing to low or excess probe concentration; respectively. Tissue fragility may also be encountered during sectioning, or due to excessive protease treatment, and/or washing steps or high hybridization temperatures. A good pre-selection (see stage B) for high quality slides prior to starting the experiment (stage C) is essential, along with the application of a specific probe in its optimal concentration thence, probe concentration should be adjusted depending on the target abundance. Therefore, this protocol could be adapted for detection of miRNAs in other plant crops (see Supplementary Table 2).

It is relevant to point out that the interpretation of *in situ* hybridization of any mRNA or miRNA that is up or down-regulated in a tissue can be a tricky matter and proper controls are very important. As described in the manuscript, it is important to accurately follow the color reaction and to stop the incubation with the developing solution before non-specific signal starts to appear on control slides. However, in some cases the abundance of miRNAs could be low and it can be in the limit of the detection sensibility of the technique. We believe that being able or not to detect a particular miRNA in galls is rather an intrinsic limit of any *in situ* hybridization procedure. Limited sensitivity of miRNA in situs will mainly depend on the appearance of non-specific signals in control tissues devoid of the target sequence.

## Conclusion

The method here described has been successfully adapted to detect miRNAs (here miR390) in particular cells and tissue types such as GCs within tomato galls and URS. The method is based on the hybridization of paraffin sections with LNA-double-labeled probes. This protocol is an important tool for the study of the cellular and tissue specific expression profiles of miRNAs that might have putative roles during gall formation and/or its maintenance. Furthermore, we think that the method can be applied to other vegetable crops that are resilient or more difficult to transform than plant models such as *Arabidopsis*.

### Timing overview of each stage of the protocol

**Table:** 

**Stage**	**Steps**	**Procedure to obtain it**	**Runtime**
A	1–24	Fixation and embedding in paraffin	8 days
B	Undetermined	Microscopy: sectioning and microscope pre-selection	~5 h per mold
C	25–47	Paraffin removal and hybridization with LNA double-labeled probes	1 day
D	48–62	Detection and development	1 day
E	63–65	Mounting and photographing	~30 min per slide

## Author contributions

FEDM performed most of the experiments related to the proof of concept and modifications from a basic protocol. MB designed the LNA probes, searched in different databases for specific probes and performed *in silico* analysis of miRNAs homologies. JdAE and CE aimed the protocol. JdAE generated and embedded tomato galls. MB, GE, and CE guided FEDM for the experiments. FEDM, JdAE, and CE wrote the manuscript. The final version was supervised by GE, CF, JdAE, and CE. All authors read and commented about details on the manuscript.

### Conflict of interest statement

The authors declare that the research was conducted in the absence of any commercial or financial relationships that could be construed as a potential conflict of interest.

## Footnotes

^1^Exiqon® website. “MRNA and lncRNA in situ hybridization.” Last modified May 2nd, 2016. http://www.exiqon.com/mrna-in-situ-hybridization

^2^Menzel Gläser® website. “Thermo Scientific™ SuperFrost Printink^©^ Objektträger.” Last modified May 2nd, 2016. http://www.menzel.de/9-0-Superfrost.html

^3^The MiRBase database. “Search miRBase.” Last modified May 2nd, 2016. http://www.mirbase.org/search.shtml

## Supplementary material

Supplementary Image 1 **Ribbons example**. Schematic representation of ISH microtome sections of specimens embedded in paraffin forming ribbons and placed on the slides. It is recommended to obtain around 12-16 ribbons per slide that will be used for each specific probe. In our hands, we obtained up to 14 ribbons maximum per slide. Thus, in total 24-32 ribbons per independent experiment are recommended per gall, and a similar number from 2 uninfected root segments (URS) for at least two probes (specific probe and negative control). Note the placement order of the ribbons in columns.

Supplementary Table 1 **Stages guidelines**. Simplified guidelines separated in different stages from (A-E) in the protocol. Details on buffers, temperature, incubation times and volumes utilized, as well as useful advices are shown.

Supplementary Table 2 **MiR390 sequences in different crop species**. BLASTN from miRBase (http://www.mirbase.org/) was used to search for miRNA homologs in different crops species matching the miR390 probe sequence (*GGCGCTATCCCTCCTGAGCTT*). The homology (score value) and significance are specified (*E*-value). Asterisks indicate 100% homology.
