# Supplementary figures and images for: A Reliable Protocol for In situ microRNAs Detection in Feeding Sites Induced by Root-Knot Nematodes
Source: Front Plant Sci. 2016 Jul 7;7:966. doi: 10.3389/fpls.2016.00966 (PMC4936241; doi:10.3389/fpls.2016.00966)

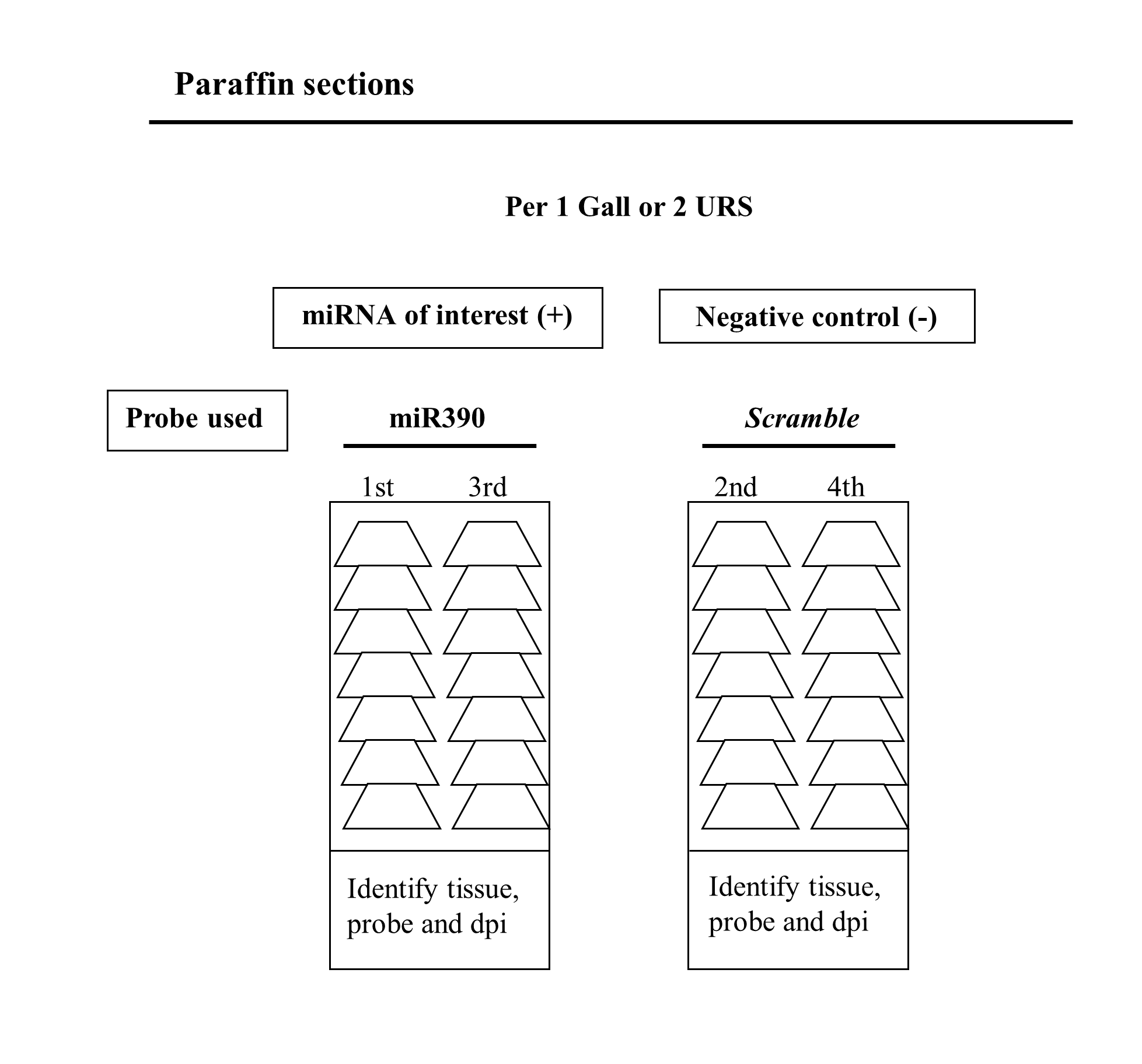

Supplement: Supplementary Image 1 — Ribbons example. Schematic representation of ISH microtome sections of specimens embedded in paraffin forming ribbons and placed on the slides. It is recommended to obtain around 12-16 ribbons per slide that will be used for each specific probe. In our hands, we obtained up to 14 ribbons maximum per slide. Thus, in total 24-32 ribbons per independent experiment are recommended per gall, and a similar number from 2 uninfected root segments (URS) for at least two probes (specific probe and negative control). Note the placement order of the ribbons in columns. [file Image1.TIF]
