# Supplementary material for: A Reliable Protocol for In situ microRNAs Detection in Feeding Sites Induced by Root-Knot Nematodes
Source: Front Plant Sci. 2016 Jul 7;7:966. doi: 10.3389/fpls.2016.00966 (PMC4936241; doi:10.3389/fpls.2016.00966)
Supplement: Supplementary Table 1 — Stages guidelines. Simplified guidelines separated in different stages from (A-E) in the protocol. Details on buffers, temperature, incubation times and volumes utilized, as well as useful advices are shown. [file Table1.DOC]

**Table S1**

**Stage A: Fixation and embedding in paraffin**

| **Day** | **Step** | **Solution** | **Temperature** | **Time** | **Volume** | **Remarks** | **Objective** |
| --- | --- | --- | --- | --- | --- | --- | --- |
| **1** | **1** | PBS 1x | Ice bed | 30 min | 1 mL/ gall or URS | Use robe, gloves and RNA sterile materials | Tissue harvesting and galls/URS collecting |
|  | **2** | Formaldehyde 4% | Ice bed | 30 s of vacuum | 1 mL/ gall or URS | Use fumehood and vacuum pump. Rest pressureless before next step | Tissue fixation |
|  | **3** | Formaldehyde 4% | Ice bed | 30 s of vacuum | 1 mL/ gall or URS | Use fumehood and vacuum pump. Rest pressureless before next step | Tissue fixation |
|  | **4** | Formaldehyde 4% | 4ºC (fridge) | 14 h | 1 mL/ gall or URS | Continuous stirring | Tissue fixation |
| **2** | **5** | PBS 1x | Ice bed | 30 min | 1 mL/ gall or URS | Continuous stirring and degassed buffer | Tissue washing |
|  | **6** | 10% ethanol/ 90% NaCl 1x | Ice bed | 1 h | 1 mL/ gall or URS | Continuous stirring and degassed buffer | Tissue dehydration |
|  | **7** | 30% ethanol/ 70% NaCl 1x | Ice bed | 1 h | 1 mL/ gall or URS | Continuous stirring and degassed buffer | Tissue dehydration |
|  | **8** | 50% ethanol/ 50% NaCl 1x | Ice bed | 1 h | 1 mL/ gall or URS | Continuous stirring and degassed buffer | Tissue dehydration |
|  | **9** | 70% ethanol/ 30% NaCl 1x | 4ºC (fridge) | Overnight | 1 mL/ gall or URS | Continuous stirring and degassed buffer | Tissue dehydration |
| **3** | **10** | 85% ethanol/ 15% NaCl 1x | 4ºC (fridge) | 1 h | 1 mL/ gall or URS | Continuous stirring and degassed buffer | Tissue dehydration |
|  | **11** | 90% ethanol/ 10% mQ water/0,1% eosin | 4ºC (fridge) | 20 min | 1 mL/ gall or URS | For a better location and inclusion | Tissue staining |
|  | **12** | 95% ethanol/ 5% mQ water | 4ºC (fridge) | 1 h | 1 mL/ gall or URS | Continuous stirring and degassed buffer | Tissue dehydration |
|  | **13** | 100% ethanol | 4ºC (fridge) | 1 h | 1 mL/ gall or URS | Continuous stirring and degassed buffer | Tissue dehydration |
|  | **14** | 100% ethanol | 4ºC (fridge) | Overnight | 1 mL/ gall or URS | Continuous stirring and degassed buffer | Tissue dehydration |
| **4** | **15** | 100% ethanol | RT | 1 h | 1 mL/ gall or URS | Continuous stirring and degassed buffer. Note that the green colour disappears | Tissue dehydration |
|  | **16** | 100% ethanol/Histoclear® | RT | 1 h | 20 mL/ 5 galls or URS | Put 3 parts of 100% ethanol and 1 of Histoclear®. Continuous stirring. Use fumehood and glass vials | Tissue inclusion |
|  | **17** | 100% ethanol/Histoclear® | RT | 1 h | 20 mL/ 5 galls or URS | Put 1 part of 100% ethanol and 1 of Histoclear®. Continuous stirring. Use fumehood and glass vials | Tissue inclusion |
|  | **18** | 100% ethanol/Histoclear® | RT | 1 h | 20 mL/ 5 galls or URS | Put 1 part of 100% ethanol and 3 of Histoclear®. Continuous stirring. Use fumehood and glass vials | Tissue inclusion |
|  | **19** | 100% Histoclear® | RT | Overnight | 20 mL/ 5 galls or URS | Continuous stirring. Use fumehood and glass vials | Tissue inclusion |
| **5** | **20** | 50% Histoclear® / 50% Paraplast® resin | 58ºC | 1 day | 20 mL/ 5 galls or URS | Refresh twice a day the solution. Leave glass vials open to favour Histoclear® evaporation. Use fumehood. | Tissue inclusion |
| **6-7** | **21** | 100% Paraplast® | 58ºC | 2 days | 20 mL/ 5 galls or URS | Refresh twice a day the solution. Use new glass vials per change | Tissue inclusion |
| **8** | **22** | 100% Paraplast® | 58ºC | 2 h | 20 mL/ 5 galls or URS per aluminium custards | Use flamed tweezers and 2 water bath plates | Tissue hardening |
|  | **23** | 100% Paraplast® | 4ºC (tap water+ice) | 5 min | Not | Note the orientation of the samples | Tissue hardening |
|  | **24** | Fixation and embedding end | RT | Years | Not | Keep it in darkness (cupboard) | Samples in Paraplast® |

**Stage B: Sectioning and microscope preselection (see Figure S1)**

**Stage** C: Deparaffinization and hybridization with LNA double labelled probes

| **Step** | **Solution** | **Temperature** | **Time** | **Volume** | **Remarks** | **Objective** |
| --- | --- | --- | --- | --- | --- | --- |
| **25** | 100% Histoclear® | RT | 4 min | 100 mL/  6 slides | Use fumehood, robe, gloves, continuous stirring and sterile glass cuvettes | Tissue dewaxing |
| **26** | 100% ethanol | RT | 1 min | 100 mL/  6 slides | Continuous stirring in sterile glass cuvettes under fume hood | Tissue hydration |
| **27** | 100% ethanol | RT | 30 s | 100 mL/  6 slides | Without stirring in sterile glass cuvettes and outside the fumehood | Tissue hydration |
| **28** | 95% ethanol/ mQ water | RT | 30 s | 100 mL/  6 slides | Without stirring in sterile glass cuvettes | Tissue hydration |
| **29** | 75% ethanol/ NaCl 1x | RT | 30 s | 100 mL/  6 slides | Without stirring in sterile glass cuvettes | Tissue hydration |
| **30** | 50% ethanol/ NaCl 1x | RT | 30 s | 100 mL/  6 slides | Without stirring in a sterile glass cuvettes | Tissue hydration |
| **31** | 30% ethanol/ NaCl 1x | RT | 30 s | 100 mL/  6 slides | Without stirring in sterile glass cuvettes | Tissue hydration |
| **32** | 10% ethanol/ NaCl 1x | RT | 30 s | 100 mL/  6 slides | Without stirring in a sterile glass cuvettes | Tissue hydration |
| **33** | PBS 1x | RT | 2 min | 100 mL/  6 slides | Without stirring in sterile glass cuvettes | Tissue hydration |
| **34** | TE buffer + protease 50 mg/mL | 37ºC | 20 min | 50 mL/  6 slides | Without stirring in sterile glass cuvettes and incubator | Eliminate RNAses |
| **35** | Glycine 0.2% | RT | 4 min | 50 mL/  6 slides | Without stirring in sterile glass cuvettes | Eliminate protease activity |
| **36** | PBS 1x | RT | 1 min | 100 mL/  6 slides | Without stirring in sterile glass cuvettes | Tissue washing |
| **37** | PBS 1x | RT | 1 min | 100 mL/  6 slides | Without stirring in sterile glass cuvettes | Tissue washing |
| **38** | 10% ethanol/ NaCl 1x | RT | 30 s | 100 mL/  6 slides | Without stirring in sterile glass cuvettes | Tissue dehydration |
| **39** | 30% ethanol/ NaCl 1x | RT | 30 s | 100 mL/  6 slides | Without stirring in sterile glass cuvettes | Tissue dehydration |
| **40** | 50% ethanol/ NaCl 1x | RT | 30 s | 100 mL/  6 slides | Without stirring in sterile glass cuvettes | Tissue dehydration |
| **41** | 75% ethanol/ NaCl 1x | RT | 30 s | 100 mL/  6 slides | Without stirring in sterile glass cuvettes | Tissue dehydration |
| **42** | 95% ethanol/ mQ water | RT | 30 s | 100 mL/  6 slides | Without stirring in sterile glass cuvettes | Tissue dehydration |
| **43** | 100% ethanol | RT | 30 s | 100 mL/  6 slides | Without stirring in sterile glass cuvettes | Tissue dehydration |
| **44** | Air drying | RT | 30 min | Not | In a sterile plastic tray and darkness | Slide drying |
| **45** | Force drying | RT | 5 s per slide | Not | On the bench, with gloves and robe | Slide preparation |
| **46** | Hybridization solution (20 nM) | 50ºC | 22 hours | 100 μl per slide | In a stainless steel box putting in an incubator. Damp paper and parafilm | MiRNA hybridization |
| **47** | Hybridization end | RT | Day after | Next step | Keep it in stainless steel box | Preparation for anti-DIG incubation and detection |

**Stage D: Detection and developing**

| **Step** | **Solution** | **Temperature** | **Time** | **Volume** | **Remarks** | **Objective** |
| --- | --- | --- | --- | --- | --- | --- |
| **48** | 0.2x SSC | Hybridization temperature (50ºC) | 1 min | 50 mL/  6 slides | Use an incubator, robe, gloves and sterile glass cuvettes | Coverslip removing |
| **49** | 0.2x SSC + 20% formamide | Hybridization temperature (50ºC) | 5 min | 50 mL/  6 slides | Use an incubator, robe, gloves and sterile glass cuvettes | Tissue washing |
| **50** | PBS 1x | RT | 2 min | 50 mL/  6 slides | Without stirring in sterile glass cuvettes | Tissue washing |
| **51** | TBS 1x | RT | 2 min | 50 mL/  6 slides | Without stirring in sterile glass cuvettes | Tissue washing |
| **52** | Blocking buffer | RT | 45 min | 1 mL/  1 slide | Without stirring, with gloves, robe and a stainless steel box | Stop hybridization reaction |
| **53** | Washing buffer | RT | 45 min | 1 mL/  1 slide | Without stirring, with gloves, robe and a stainless steel box | Tissue washing |
| **54** | Anti-DIG buffer | 37ºC | 1 h 50 min | 1 mL/  1 slide | Use an incubator, robe, gloves and a stainless steel box | MiRNA detection |
| **55** | Washing buffer | RT | 15 min | 1 mL/  1 slide | On the bench, with gloves, robe and a stainless steel box | Tissue washing |
| **56** | Washing buffer | RT | 15 min | 1 mL/  1 slide | On the bench, with gloves, robe and a stainless steel box | Tissue washing |
| **57** | TBS 1x | RT | 2 min | 50 mL/  6 slides | Without stirring in sterile glass cuvettes and darkness | Tissue washing |
| **58** | TN 1x | RT | 5 min | 50 mL/  6 slides | Without stirring in sterile glass cuvettes and darkness | Raising tissue pH |
| **59** | NBT/BCIP | RT | 22 hours | 50 mL/  6 slides | Without stirring in sterile glass cuvettes and darkness | Tissue developing |
| **60** | TE 1x | RT | 5 min | 50 mL/  6 slides | Without stirring in a sterile glass cuvettes | Stop developing reaction |
| **61** | Air drying | RT | 1 h to overnight | Slides | In a sterile plastic tray and darkness | Slide drying |
| **62** | Detection and developing end | RT | Next step | Slides | Keep it in a sterile plastic tray | Samples ready for mounting |

**Stage E:** Mounting and photographing

| **Step** | **Solution** | **Temperature** | **Time** | **Volume** | **Remarks** | **Objective** |
| --- | --- | --- | --- | --- | --- | --- |
| **63** | Mounting medium | RT | 2 min/slide | 1 mL/  1 slide | With a pipette, filter tips and coverslips | Slide mounting |
| **64** | Air drying | RT | 1 h to overnight | Not | In a sterile plastic tray and darkness | Slide drying |
| **65** | Microscope photographing | RT | Undetermined | Not | Take pictures on bright field optics | Results observation and final imaging |
